# Noradrenaline Modulates the Membrane Potential and Holding Current of Medial Prefrontal Cortex Pyramidal Neurons via β_1_-Adrenergic Receptors and HCN Channels

**DOI:** 10.3389/fncel.2017.00341

**Published:** 2017-11-02

**Authors:** Katarzyna Grzelka, Przemysław Kurowski, Maciej Gawlak, Paweł Szulczyk

**Affiliations:** Laboratory of Physiology and Pathophysiology, Centre for Preclinical Research and Technology, Medical University of Warsaw, Warsaw, Poland

**Keywords:** pyramidal neurons, prefrontal cortex, adrenergic receptors, HCN channel, βγ subunit, membrane potential, holding current, rats

## Abstract

The medial prefrontal cortex (mPFC) receives dense noradrenergic projections from the locus coeruleus. Adrenergic innervation of mPFC pyramidal neurons plays an essential role in both physiology (control of memory formation, attention, working memory, and cognitive behavior) and pathophysiology (attention deficit hyperactivity disorder, posttraumatic stress disorder, cognitive deterioration after traumatic brain injury, behavioral changes related to addiction, Alzheimer’s disease and depression). The aim of this study was to elucidate the mechanism responsible for adrenergic receptor-mediated control of the resting membrane potential in layer V mPFC pyramidal neurons. The membrane potential or holding current of synaptically isolated layer V mPFC pyramidal neurons was recorded in perforated-patch and classical whole-cell configurations in slices from young rats. Application of noradrenaline (NA), a neurotransmitter with affinity for all types of adrenergic receptors, evoked depolarization or inward current in the tested neurons irrespective of whether the recordings were performed in the perforated-patch or classical whole-cell configuration. The effect of noradrenaline depended on β_1_- and not α_1_- or α_2_-adrenergic receptor stimulation. Activation of β_1_-adrenergic receptors led to an increase in inward Na^+^ current through hyperpolarization-activated cyclic nucleotide-gated (HCN) channels, which carry a mixed Na^+^/K^+^ current. The protein kinase A- and C-, glycogen synthase kinase-3β- and tyrosine kinase-linked signaling pathways were not involved in the signal transduction between β_1_-adrenergic receptors and HCN channels. The transduction system operated in a membrane-delimited fashion and involved the βγ subunit of G-protein. Thus, noradrenaline controls the resting membrane potential and holding current in mPFC pyramidal neurons through β_1_-adrenergic receptors, which in turn activate HCN channels via a signaling pathway involving the βγ subunit.

## Introduction

Cortical neurons, including medial prefrontal cortex (mPFC) neurons, receive dense noradrenergic innervation from the locus coeruleus (Branchereau et al., 1996; Berridge and Waterhouse, 2003; Agster et al., 2013; Chandler et al., 2014). Noradrenaline (NA) released in the cortex controls memory formation, attention, working memory, and cognitive behaviors (Sara, 2009; Chamberlain and Robbins, 2013). Impairment of noradrenergic mPFC neuronal control occurs in multiple neuropsychiatric disorders, e.g., attention deficit hyperactivity disorder (Sheridan et al., 2010), posttraumatic stress disorder (Fitzgerald et al., 2015), traumatic brain injury-induced cognitive deterioration (Jenkins et al., 2016), Alzheimer’s disease (Gannon et al., 2015), depression (Lemogne et al., 2009; Stone et al., 2011), and behavioral changes related to addiction (Schmidt and Weinshenker, 2014).

NA-related control of behavior depends, at least in part, on the modulation of neuronal ion channels, which in turn alter neuronal activity (Wang and McCormick, 1993; Ishibashi et al., 2003; Li and van den Pol, 2005; Zhang et al., 2013). Arnsten (2009) introduced the working hypothesis that adrenergic-dependent behavioral changes depend on the noradrenergic control of the working memory process. She suggested that low levels of NA in the mPFC optimize working memory function, while high levels weaken working memory and lead to the behavioral impairment found in neuropsychiatric disorders. The proposed functional substrate of working memory is a series of action potentials at the peak of prolonged depolarizations (“up-states”) found in layer V mPFC pyramidal neurons (O’Donnell, 2008). If NA influences the working memory process, it probably does so by activating adrenergic receptors and modulating ion channels, producing a prolonged depolarization (an up-state) in pyramidal neurons with a series of action potentials at its peak (Marzo et al., 2009; Schmidt and Weinshenker, 2014).

NA released from synaptic endings may elicit its effects via activation of three classes of adrenergic G-protein-coupled receptors, α_1_, α_2_, and β. All adrenergic receptors are present in mPFC pyramidal neurons (α_1_, Santana et al., 2013; α_2_, Andrews and Lavin, 2006; Carr et al., 2007 and β, Ji et al., 2008; Zhou et al., 2013). These receptors may control cellular effectors by modulating transduction systems associated with a variety of intracellular signaling pathways, e.g., protein kinase A or C (Benovic et al., 1988; Cotecchia et al., 1990; Nishizuka, 1992; Simonds, 1999; Koshimizu et al., 2002; Hein, 2006; Ramos and Arnsten, 2007), glycogen synthase kinase-3β (Zhang et al., 2011; Daniels et al., 2012; Morioka et al., 2014; Xing et al., 2016), and tyrosine kinase (Benovic, 2002; Huang et al., 2014). Adrenergic receptors may also control ion channels in a membrane-delimited fashion by activating G-protein βγ subunits (Daaka et al., 1997; Lin and Smrcka, 2011).

The effects of adrenergic receptor stimulation on different features of mPFC neurons have been investigated (Kawaguchi and Shindou, 1998; Devilbiss and Waterhouse, 2000; Dembrow et al., 2010; Wang et al., 2013). However, the mechanism by which NA controls the membrane potential and holding current in mPFC pyramidal neurons remains unclear. Stimulation of adrenergic receptors leads to changes in the membrane potential level or holding current in cortical neurons, corresponding to either depolarization or hyperpolarization and evoking changes in neuronal excitability (McCormick et al., 1993; Wang and McCormick, 1993; Mueller et al., 2008). Wang and McCormick (1993) proposed that NA changes firing rates and evokes depolarization of cortical neurons by activating α_1_-adrenergic receptors, resulting in the subsequent inhibition of both voltage-independent and voltage- and Ca^++^-sensitive K^+^ currents. Activation of α_2_-adrenergic receptors evokes hyperpolarization and increases the excitability of mPFC pyramidal neurons by inhibiting the *I*_h_ current (Carr et al., 2007; Zhang et al., 2013). In turn, Mueller et al. (2008) proposed that NA increases the excitability of infralimbic PFC pyramidal neurons through stimulation of β-adrenergic receptors; however, the ionic mechanism was not investigated in their study. Thus far, there is no agreement on the mechanism by which the membrane potential of mPFC pyramidal neurons is controlled by NA. Therefore, the aim of our study was to clarify which adrenergic receptor controls the resting membrane potential and holding current in synaptically isolated layer V mPFC pyramidal neurons and to provide a detailed mechanism underpinning the action of NA, including the cellular effector and transduction pathway involved.

## Materials and Methods

All experimental procedures conformed to the institutional and international guidelines on the ethical use of animals and were approved by the Second Local Ethics Committee for Animal Experimentation in Warsaw (Decision 71/2014).

### Brain Slice Preparation

Medial prefrontal cortex slices were prepared from young 18- to 22-day-old male Wistar rats provided by the local animal house. The animals were decapitated, and their brains were removed and immersed in ice-cold (0–4°C), oxygenated solution containing the following components (mM): NaCl (125), NaHCO_3_ (25), KCl (3), NaH_2_PO_4_ (1.25), CaCl_2_ (0.5), MgCl_2_ (6), and glucose (25) (pH 7.4, osmolality 280 mOsm/kg H_2_O). A vibratome (Vibratome Line, Leica VT1200S, Nussloch, Germany) was used to cut 300-μm- and 150-μm-thick slices for electrophysiology and confocal microscopy, respectively. The slices were then transferred to a pre-chamber with regular artificial cerebrospinal fluid (ACSF) containing the following components (mM): NaCl (125), NaHCO_3_ (25), KCl (3), NaH_2_PO_4_ (1.25), CaCl_2_ (2), MgCl_2_ (1), and glucose (25) (pH 7.4, osmolality 320-330 mOsm/kg H_2_O). The solution was bubbled with 95% O_2_ and 5% CO_2_ and heated to 33°C. The slices were incubated in warm ACSF for 15 min and then left to recover at room temperature for at least 1 h prior to recordings.

### Perforated-Patch and Classical Whole-Cell Recordings

**FIGURE 1:**
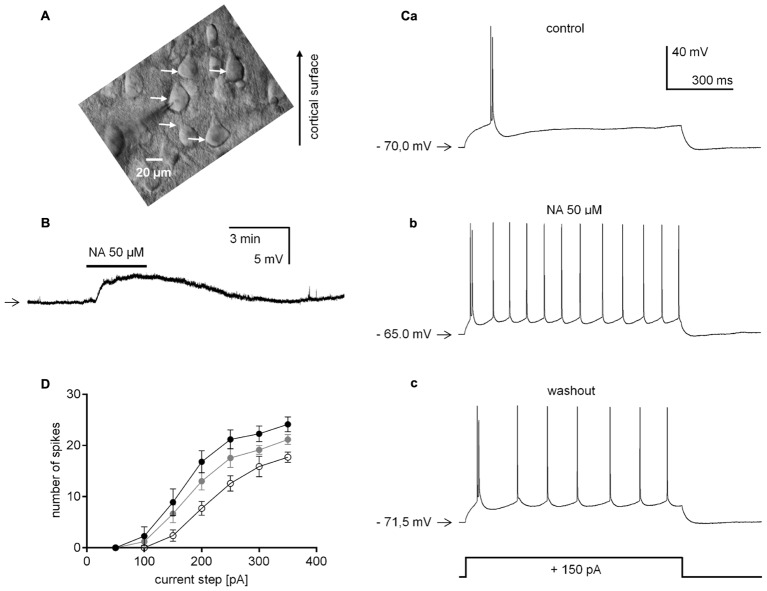
Effect of noradrenaline (NA) on the neuronal excitability of layer V medial prefrontal cortex (mPFC) pyramidal neurons in the absence of TTX. **(A)** IR-DIC image of a typical PFC slice with layer V pyramidal neurons (marked with white arrows). **(B)** Membrane potential depolarization evoked by bath application of NA (50 μM) recorded in the current-clamp classical whole-cell configuration. **(C)** Representative traces obtained from one neuron showing the response to a depolarizing current step (+150 pA, 1000 ms) before (control, **a**), during (NA 50 μM, **b**) and after NA bath application (washout, **c**). **(D)** Mean number of spikes evoked by depolarizing current steps (from +50 to +350 pA in 50 pA increments) before (control, open circles), during (NA 50 μM, black circles) and after (washout, gray circles) NA bath application. Horizontal arrows shown in this and other figures indicate the resting membrane potential or control holding current level. Continuous horizontal bars above the recording traces indicate the bath application of the agonists in this and other figures.

For the experiment, the slices were placed in a superfusion recording chamber (RC-24E, Warner Instruments, LLC, Hamden, MA, United States) on the stage of an upright Nikon microscope (Eclipse E600FN, Nikon Instech Co., Ltd., Japan). The slices were continuously perfused with ACSF (the same as above) at a rate of 2–3 ml/min and maintained at 34°C using a TC-324B temperature controller (Warner Instruments). Recordings were obtained from infralimbic and prelimbic mPFC pyramidal neurons located in layer V (600–800 μM from the cortical surface). The neurons were visualized using infra-red differential interference contrast (IR-DIC) microscopy with a 40x water immersion objective, video imaging camera (C7500-50, Hamamatsu Photonics K.K, Japan) and camera controller (C2741-62, Hamamatsu Photonics K.K). The neurons were recognized by their characteristic triangular soma shape and prominent apical dendrite (**Figure 1A**).

Electrophysiological recordings were obtained with a Multiclamp 700A amplifier, Digidata 1550B converter and pClamp 10.6 software (Molecular Devices, Sunnyvale, CA, United States), sampled at 20 kHz, and filtered at 2 kHz.

Measurements were performed in the presence of 0.5 μM tetrodotoxin (TTX) and the gamma-aminobutyric acidergic (GABAergic) and glutamatergic transmission blockers, including 50 μM picrotoxin, 10 μM 6,7-dinitroquinoxaline-2,3-dione (DNQX) and 50 μM AP-5. In some experiments, the concentration of Na^+^ ions in the extracellular solution was decreased from 151.25 to 26.25 mM by replacing NaCl (125 mM) with an equimolar concentration of choline chloride (125 mM). Data presented in **Figures 1B–D** were obtained in the presence of GABAergic and glutamatergic transmission blockers but without TTX in the extracellular solution.

Patch pipettes were pulled from borosilicate glass capillaries (OD 1.5 mm, I.D. 0.86 mm; Harvard Apparatus, Edenbridge, United Kingdom) on a horizontal puller (P-97, Sutter Instruments, Novato, CA, United States). The pipette tip resistance was 3–5 MΩ. The pipette offset potential was adjusted with the amplifier.

For recordings performed in the gramicidin perforated-patch mode, the pipettes were filled with an internal solution containing the following components (mM): potassium gluconate (105), KCl (20), HEPES-Na^+^ (10), and EGTA (0.1) (pH 7.25 adjusted with KOH, osmolality 280 mOsm/kg H_2_O). Gramicidin was dissolved in dimethyl sulfoxide (DMSO), resulting in a 10 mg/ml stock solution that was added to the internal solution at a final concentration of 20–25 μg/ml. New stock and internal solutions were prepared every 2–3 h, as gramicidin loses activity over time. After a gigaseal was obtained, the access resistance gradually decreased, indicating the progression of membrane perforation. Once the access resistance was stable, usually 10–30 min after gigaseal formation, the recording started. The access resistance was controlled at regular intervals; if it rapidly decreased, indicating a spontaneous rupture of the membrane patch, the recording was discarded. After each recording in perforated-patch mode, the membrane was ruptured by suction, followed by a marked decrease in access resistance (Akaike and Harata, 1994; Akaike, 1999).

For measurements performed in the classical whole-cell configuration, the pipettes were filled with an internal solution containing the following components (mM): potassium gluconate (110); KCl (20); MgCl_2_ (2); ATP_2_Na (2), GTPNa (0.4), NaCl (5), HEPES (10), and EGTA (0.5) (pH 7.4 adjusted with KOH, osmolality 280 mOsm/kg H_2_O). After gigaseal formation, the cell membrane was ruptured by suction.

Only one neuron in each slice was exposed to a tested compound a single time, and the slice was replaced after one test on a single cell was performed. For example, in slices obtained from one rat, 7 neurons from 7 slices were usually tested. From these slices, three cells (in three slices) were exposed to the adrenergic agonist alone (control), and four cells (in four slices) were examined using agonist application together with application of a blocker or inhibitor. The effect of the same blocker or inhibitor was tested on slices obtained from at least three rats. The results of the application of the agonist in the presence of a blocker or inhibitor were compared to control results recorded from neurons in slices obtained from the same rats. All agonists were applied for 3 min. Blockers or inhibitors were included in the bath (extracellular solution) for at least 10 min before agonist application, during the 3-min agonist application and at least 10 min after agonist application. Occasionally, as indicated in the text, compounds were included in the pipette solution (intracellular solution), or the slices were preincubated with the tested compound.

Recordings usually began >5 min after obtaining access to the cell. When the GRK2i polypeptide was applied to the pipette solution, the electrophysiological recordings started >50 min after cell membrane rupture to allow the compound to move from the pipette into the cell interior. When other inhibitors of the transduction systems were added to the pipette solution, the electrophysiological recordings began >15 min after access to the cell was obtained. The access resistance was regularly monitored.

The holding current in the voltage-clamp configuration (Goodfellow et al., 2009; Li et al., 2017) was adjusted so that the neurons were held at their physiological membrane potential measured in the current-clamp configuration.

### Confocal Microscopy

The 150-μm-thick prefrontal cortex slices were fixed in 4% paraformaldehyde in PBS (at 4°C) for 6 h. The free-floating sections were blocked with 5% goat serum in PBS and incubated with the rabbit anti-β1-adrenergic receptor antibody (1:200, Abcam, catalog number: ab3442) overnight at 4°C. Unbound antibodies were washed out with PBS, and the Cy3-conjugated goat anti-rabbit (1:200, Jackson ImmunoResearch, catalog number: 111-165-144) secondary antibody was applied at room temperature for 2 h. Next, the sections were washed with PBS (four times for 5 min each), mounted in medium (Vectashield with DAPI, Vector Laboratories, catalog number H-1200) and coverslipped. The specificity of the applied primary antibodies (Abcam, catalog number: ab3442) was recently documented (Lv et al., 2016; Tyurin-Kuzmin et al., 2016; Huang et al., 2017; Kim et al., 2017; Mesquita et al., 2017). Immunofluorescence measurements were performed with a confocal laser scanning microscope (FV1000, Olympus, objectives 10× and 60×, image 1024 pixels × 1024 pixels).

### Drugs

ZD 7288, daidzein and picrotoxin were purchased from HelloBio (Bristol, United Kingdom); NA bitartrate, yohimbine hydrochloride, metoprolol tartrate, and DNQX from Abcam (Cambridge, United Kingdom); TDZD-8, gramicidin, and choline chloride from Sigma–Aldrich (St. Louis, MO, United States); genistein and AP-5 from Alomone Labs (Jerusalem, Israel); dobutamine hydrochloride from Sandoz GmbH (Kundl, Austria); and TTX from Latoxan (Valence, France). All other compounds were purchased from Bio-Techne (Abingdon, United Kingdom).

The compounds were dissolved at the specified final concentration in ACSF and added to the bath (VC-6 six-channel valve controller, Warner Instruments) or pipette solution when indicated. To protect the compounds from degradation, solutions containing NA, dobutamine, isoproterenol, gallein, TDZD-8 and genistein were freshly prepared before the application and stored in the dark.

If a compound was dissolved in DMSO, then the same concentration of DMSO was added to the extracellular or intracellular solution for the control recordings.

### Data Analysis

The data were analyzed using GraphPad Prism 7 software. Unpaired or paired two-tailed Student’s *t*-test and repeated measures one-way ANOVA with Tukey’s multiple comparisons test were used for statistical analysis, as appropriate. Differences were considered statistically significant at *p* < 0.05. The effects of agonists in the presence of different blockers or inhibitors were compared to control measurements (when the agonist was applied alone) performed on different neurons in different slices isolated from the same rats. To test whether the membrane potential (or number of spikes) changed significantly in the tested condition, we used a two-tailed one-sample *t*-test with the null hypothesis stating that there is no difference in the membrane potential (number of spikes) between control and tested conditions. All results are presented as the mean ± SEM.

The membrane potential and holding current traces shown in the figures were adequately filtered off line (Boxcar filter from Clampfit).

## Results

### The Effects of Noradrenaline on the Membrane Potential and Holding Current in Pyramidal Neurons

Data presented in **Figure 1** were obtained in the absence of TTX and in the presence of GABAergic and glutamatergic blockers in the extracellular solution. Application of NA (50 μM, 3 min) to the bath significantly depolarized the membrane potential of layer V mPFC pyramidal neurons by 3.4 ± 0.3 mV (*n* = 11, *p* < 0.0001, one-sample *t*-test, **Figure 1B**). To test pyramidal neuron excitability, rectangular current steps lasting 1000 ms in 50-pA increments were applied before NA application, during NA application and during NA washout. **Figure 1C** demonstrates the action potentials evoked by a depolarizing 1000-ms, 150-pA rectangular current step before (**Figure 1Ca**) and during (**Figure 1Cb**) NA bath application and during NA washout (**Figure 1Cc**) in a single pyramidal neuron.

To test the effect of NA on the number of spikes induced by different depolarizing steps, we used a repeated measures one-way ANOVA (*p* < 0.004, followed by Tuckey’s multiple comparisons test). The mean number of spikes at each depolarizing current step above 50 pA was higher during NA application (*n* = 10, *p* < 0.01, Tuckey’s multiple comparisons test, **Figure 1D** black circles) than in the control condition before NA application (*n* = 10, **Figure 1D** open circles). During washout (*n* = 9, **Figure 1D** gray circles), there were fewer spikes at each depolarizing current step above 50 pA than during NA application (*p* < 0.01, Tuckey’s multiple comparisons test).

The results described below were obtained in the presence of GABAergic and glutamatergic blockers in the extracellular solution, as well as TTX, which was added to block any spontaneous activity in the slice.

The resting membrane potential levels recorded in the perforated-patch (-67.7 ± 0.4, *n* = 122) and classical whole-cell (-67.2 ± 0.2, *n* = 303) configurations were not significantly different (*p* = 0.1735, unpaired *t*-test) in synaptically isolated, layer V mPFC pyramidal neurons in slices.

**FIGURE 2:**
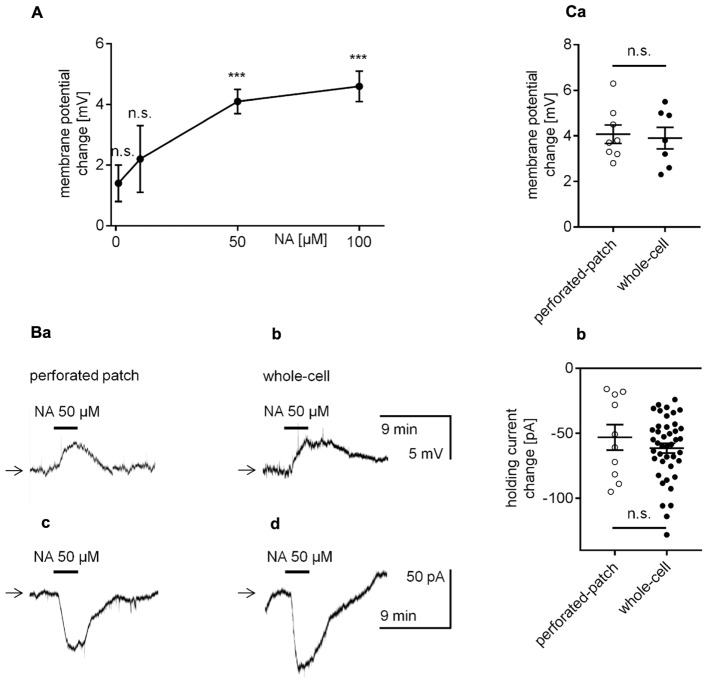
Effect of NA on the membrane potential and holding current in layer V mPFC pyramidal neurons. **(A)** Concentration-response curve for the effect of NA on the amplitude of the membrane potential depolarization. **(B)** Membrane potential depolarization evoked by bath application of NA (50 μM) recorded in the current-clamp perforated-patch **(a)** and classical whole-cell **(b)** configurations. Inward current evoked by bath application of NA (50 μM) recorded in the voltage-clamp perforated-patch **(c)** and classical whole-cell **(d)** configurations. **(C)** Depolarization amplitudes evoked by NA (50 μM) recorded in the current-clamp perforated-patch and classical whole-cell configurations **(a)**. Inward current amplitudes evoked by NA (50 μM) recorded in the voltage-clamp perforated-patch and classical whole-cell configurations **(b)**; ^∗∗∗^*p* < 0.001; n.s., non-significant. In this **(Ca,b)** and other figures, amplitudes of membrane potentials are shown as M ± SE and the distribution of individual measurements.

The effect of NA on the membrane potential in mPFC pyramidal neurons was tested in the perforated-patch configuration. Application of NA (3 min, 1–100 μM) to the bath evoked a membrane potential depolarization, which recovered with washout (**Figures 2A,Ba**). The change in the membrane potential was not significant at 1 μM (1.4 ± 0.6 mV, *n* = 5, *p* = 0.0754, one-sample *t*-test) or 10 μM NA (2.2 ± 1.1 mV, *n* = 9, *p* = 0.0907, one-sample *t*-test); however, the membrane potential significantly increased with the addition of 50 μM (4.1 ± 0.4 mV, *n* = 8, *p* < 0.0001, one-sample *t*-test) and 100 μM NA (4.6 ± 0.5 mV, *n* = 10, *p* < 0.0001, one-sample *t*-test). In subsequent experiments, 50 μM NA was applied.

The amplitude of the NA-related depolarization was not significantly different between recordings in the perforated-patch (4.1 ± 0.4 mV, *n* = 8, **Figures 2Ba,Ca**) or classical whole-cell configuration (3.9 ± 0.5 mV, *n* = 7, *p* = 0.7825, unpaired *t*-test, **Figures 2Bb,Ca**).

Bath application of NA evoked a significant holding current change in all tested pyramidal neurons when recorded in the perforated-patch (-53.1 ± 9.8 pA, *n* = 10, *p* = 0.0004, one-sample *t*-test, **Figures 2Bc,Cb**) or classical whole-cell configuration (-61.4 ± 3.8 pA, *n* = 42, *p* < 0.0001, one-sample *t*-test, **Figures 2Bd,Cb**). The amplitude of the inward current was likewise not significantly different between recordings performed in both configurations (*p* = 0.3641, unpaired *t*-test, **Figures 2Cb**).

Thus, NA depolarized mPFC pyramidal neurons and evoked an inward current.

### Identification of the Adrenergic Receptor Responsible for Noradrenaline-Dependent Depolarization and Inward Current in Pyramidal Neurons

**FIGURE 3:**
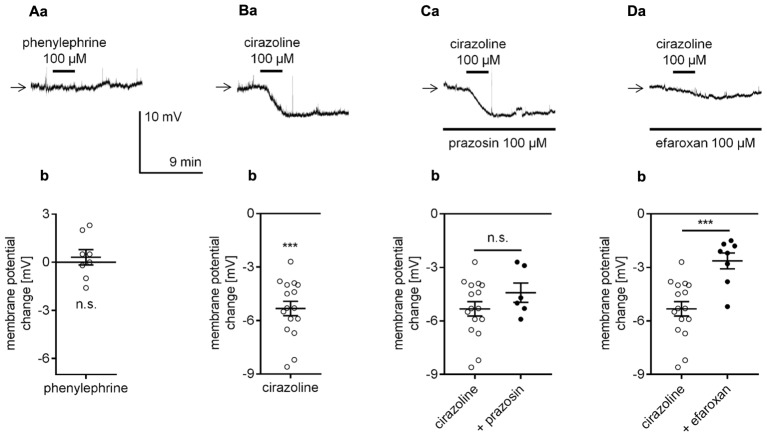
Effect of α_1_-adrenergic receptor stimulation on the membrane potential of layer V mPFC pyramidal neurons. **(A)** Membrane potential recorded during bath application of phenylephrine (100 μM, **a**). Amplitude of the membrane potential change evoked by 100 μM phenylephrine (phenylephrine, **b**). **(B)** Membrane potential change evoked by bath application of cirazoline (100 μM, **a**). Amplitude of the membrane potential change evoked by 100 μM cirazoline (cirazoline, **b**). **(C)** Membrane potential change evoked by bath application of cirazoline (100 μM) in the presence of prazosin (100 μM in the bath, **a**). Amplitude of the membrane potential change evoked by 100 μM cirazoline alone (cirazoline) and 100 μM cirazoline in the presence of 100 μM prazosin (+ prazosin, **b**). **(D)** Membrane potential change evoked by bath application of cirazoline (100 μM) in the presence of efaroxan (100 μM in the bath, **a**). Amplitude of the membrane potential change evoked by 100 μM cirazoline alone (cirazoline) and 100 μM cirazoline in the presence of 100 μM efaroxan (+ efaroxan, **b**); ^∗∗∗^*p* < 0.001; n.s., non-significant. Continuous horizontal bars below the recording traces indicate the bath/extracellular solution presence of the compounds in this and other figures.

NA has an affinity for all types of adrenergic receptors (α_1_, α_2_, and β). We assessed whether activation of each type of adrenergic receptor was able to mimic the effect of NA. First, the effect of α_1_-adrenergic receptor stimulation on the membrane potential was tested in the perforated-patch mode. Application of a selective α_1_-adrenergic receptor agonist phenylephrine (100 μM) at a dose shown to affect mPFC properties (Kobayashi, 2007; Zhang et al., 2013; Luo et al., 2015a,b) did not alter the resting membrane potential level (0.3 ± 0.5 mV, *n* = 8, *p* = 0.5312, one-sample *t*-test, **Figures 3Aa,b**). Cirazoline (100 μM), another widely used α_1_-adrenergic receptor agonist (Croce et al., 2003; Imbery et al., 2008), failed to mimic NA-dependent depolarization. Conversely, bath application of cirazoline evoked hyperpolarization (-5.3 ± 0.4 mV, *n* = 16, *p* < 0.0001, one-sample *t*-test, **Figures 3Ba,b**). The amplitude of the cirazoline-dependent hyperpolarization in the presence of an α_1_-adrenergic receptor blocker prazosin in the bath (100 μM, Kobayashi, 2007; Luo et al., 2014, **Figures 3Ca,b**) was not significantly different than that in the presence of cirazoline alone (-4.4 ± 0.5 mV, *n* = 6, *p* = 0.2344, unpaired *t*-test). Cirazoline can activate imidazoline receptors (Chung et al., 2010). Therefore, the effect of cirazoline on the membrane potential was tested in the presence of an imidazoline receptor antagonist efaroxan (100 μM). In the presence of efaroxan, the amplitude of the cirazoline-related hyperpolarization was significantly smaller (-2.6 ± 0.4 mV, *n* = 8, *p* = 0.0005, unpaired *t*-test, **Figures 3Da,b**) than the amplitude with cirazoline alone. Neither prazosin (*n* = 16, *p* = 0.5161) nor efaroxan (*n* = 8, *p* = 0.0727) changed the control membrane potential when applied to the extracellular solution alone (data not shown). Based on these results, we suggest that α_1_-adrenergic receptors do not control the resting membrane potential in layer V mPFC pyramidal neurons.

**FIGURE 4:**
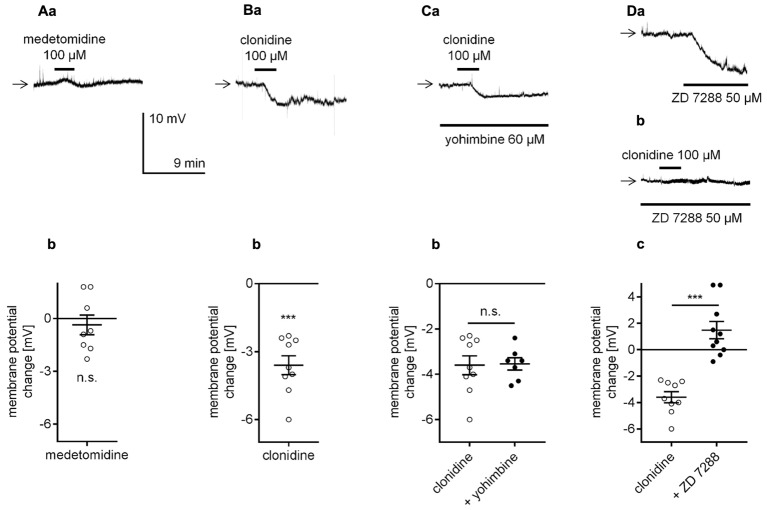
Effect of α_2_-adrenergic receptor stimulation on the membrane potential of layer V mPFC pyramidal neurons. **(A)** Membrane potential recorded during bath application of medetomidine (100 μM, **a**). Amplitude of the membrane potential change evoked by 100 μM medetomidine (medetomidine, **b**). **(B)** Membrane potential change evoked by bath application of clonidine (100 μM, **a**). Amplitude of the membrane potential change evoked by 100 μM clonidine (clonidine, **b**). **(C)** Membrane potential change evoked by bath application of clonidine (100 μM) in the presence of yohimbine (60 μM in the bath, **a**). Amplitude of the membrane potential change evoked by 100 μM clonidine alone (clonidine) and 100 μM clonidine in the presence of 60 μM yohimbine (+ yohimbine, **b**). **(D)** Membrane potential changes evoked by bath application of ZD 7288 (50 μM) alone **(a)** and bath application of clonidine (100 μM) in the presence of ZD 7288 (50 μM in the bath, **b**). Amplitude of the membrane potential change evoked by 100 μM clonidine alone (clonidine) and 100 μM clonidine in the presence of 50 μM ZD 7288 (+ ZD 7288, **c**); ^∗∗∗^*p* < 0.001; n.s., non-significant.

Next, the effect of α_2_-adrenergic receptor stimulation on resting membrane potential was tested in mPFC pyramidal neurons. For that purpose, a selective α_2_-adrenergic receptor agonist was applied to the bath while the membrane potential was recorded in the perforated-patch configuration. Application of the selective α_2_-adrenergic receptor agonist, medetomidine (100 μM, Scheibner et al., 2001; Albarrán-Juárez et al., 2009; Giovannitti et al., 2015), did not evoke any significant changes in the resting membrane potential of the tested neurons (-0.4 ± 0.6 mV, *n* = 8, *p* = 0.5376, one-sample *t*-test, **Figures 4Aa,b**). Clonidine (100 μM), another agonist, is widely used to stimulate α_2_-adrenergic receptors (Carr et al., 2007; Wolff et al., 2007; Dembrow et al., 2010; Cathel et al., 2014). In our study, clonidine bath application hyperpolarized the membrane potential (-3.6 ± 0.4 mV, *n* = 9, *p* < 0.0001, one-sample *t*-test, **Figures 4Ba,b**). Therefore, compared with bath application of NA, which evoked depolarization (**Figures 2Ba,Ca**), bath application of clonidine had an opposite effect on the membrane potential. Clonidine-dependent hyperpolarization was not diminished in the presence of yohimbine (60 μM), an α_2_-adrenergic receptor blocker (-3.5 ± 0.3 mV, *n* = 7, *p* = 0.9160, unpaired *t*-test, **Figures 4Ca,b**), in the bath, suggesting that clonidine did not act through α_2_-adrenergic receptors. Yohimbine alone did not evoke any significant changes in the membrane potential (*n* = 7, *p* = 0.1427, data not shown). Knaus et al. (2007) showed that clonidine may exert its effect via direct inhibition of hyperpolarization-activated cyclic nucleotide-gated (HCN) channels. For this reason, we tested the effect of clonidine in the bath presence of a selective HCN channel blocker ZD 7288 (50 μM, Gasparini and DiFrancesco, 1997; Day et al., 2005; Li et al., 2010). The addition of ZD 7288 to the extracellular solution hyperpolarized the membrane potential when compared to the mean resting membrane potential in the control condition (-76.4 ± 1.2, *n* = 31 in the presence of ZD 7288 50 μM and -68.9 ± 0.8 mV, *n* = 26 in the control, *p* < 0.0001, unpaired *t*-test, **Figure 4Da**). When the hyperpolarized membrane potential in the presence of ZD 7288 was stable, application of clonidine (100 μM) did not evoke further hyperpolarization in the tested neurons (+1.5 ± 0.7 mV, *n* = 10, *p* < 0.0001, unpaired *t*-test, **Figures 4Db,c**), suggesting that clonidine hyperpolarizes the membrane via direct inhibition of HCN channels. Therefore, our results suggest that α_2_-adrenergic receptors do not control the resting membrane potential in pyramidal neurons.

**FIGURE 5:**
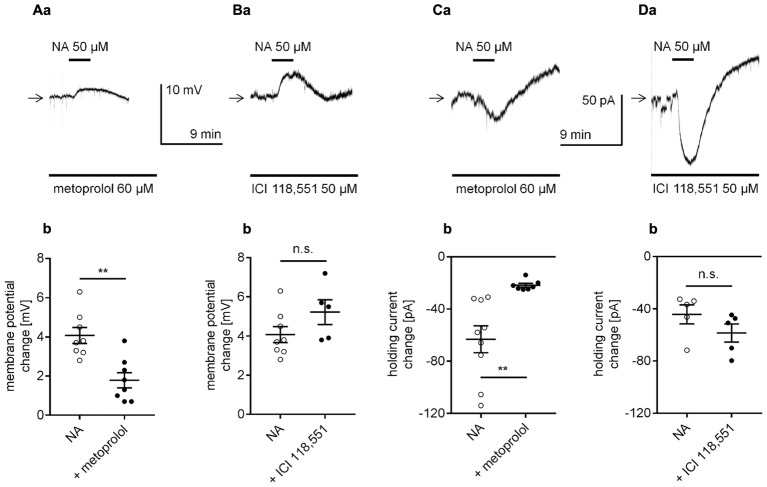
Effect of selective β-adrenergic receptor blockers on NA-induced depolarization **(A,B)** and the inward current **(C,D)** in layer V mPFC pyramidal neurons. **(A)** Depolarization evoked by bath application of NA (50 μM) in the presence of the selective β_1_-adrenergic receptor blocker metoprolol (60 μM in the bath, **a**). Amplitude of the membrane potential change evoked by 50 μM NA alone (NA) and 50 μM NA in the presence of 60 μM metoprolol (+ metoprolol, **b**). **(B)** Depolarization evoked by bath application of NA (50 μM) in the presence of the selective β_2_-adrenergic receptor blocker ICI 118551 (50 μM in the bath, **a**). Amplitude of the membrane potential change evoked by 50 μM NA alone (NA) and 50 μM NA in the presence of 50 μM ICI 118551 (+ ICI 118551, **b**). **(C)** Inward current evoked by bath application of NA (50 μM) in the presence of the selective β_1_-adrenergic receptor blocker metoprolol (60 μM in the bath, **a**). Amplitude of the holding current change evoked by 50 μM NA alone (NA) and 50 μM NA in the presence of 60 μM metoprolol (+ metoprolol, **b**). **(D)** Inward current evoked by bath application of NA (50 μM) in the presence of the selective β_2_-adrenergic receptor blocker ICI 118551 (50 μM in the bath, **a**). Amplitude of the holding current change evoked by 50 μM NA alone (NA) and 50 μM NA in the presence of 50 μM ICI 118551 (+ ICI 118551, **b**); ^∗∗^*p* < 0.01; n.s., non-significant.

To determine whether NA depolarized the membrane potential via β-adrenergic receptor stimulation, we applied NA to the bath in the presence of selective β-adrenergic receptor antagonists, and the membrane potential was recorded in the perforated-patch configuration. In the presence of the selective β_1_-adrenergic receptor blocker metoprolol (60 μM, Oshima et al., 2014), the amplitude of the NA-dependent depolarization was significantly smaller (1.8 ± 0.4 mV, *n* = 8, *p* = 0.0011, unpaired *t*-test, **Figures 5Aa,b**) than in the absence of the blocker (4.1 ± 0.4 mV, *n* = 8). In the presence of the selective β_2_-adrenergic blocker ICI 118,551 (50 μM, Rankovic et al., 2011; Zhou et al., 2013), the amplitude of the NA-dependent depolarization was not significantly different (5.2 ± 0.6 mV, *n* = 5, *p* = 0.1364, unpaired *t*-test, **Figures 5Ba,b**) from that of the depolarization induced by NA alone (4.1 ± 0.4 mV, *n* = 8). Neither metoprolol (*n* = 19, *p* = 0.1541) nor ICI 118,551 (*n* = 11, *p* = 0.5450) significantly changed the membrane potential level when applied alone (data not shown).

Additionally, in the classical whole-cell configuration, the NA-dependent inward current was markedly smaller in the presence of the selective β_1_-adrenergic receptor blocker metoprolol in the bath (-21.8 ± 1.5 pA, *n* = 7, *p* = 0.0035, unpaired *t*-test, **Figures 5Ca,b**) than in the presence of NA in the absence of the blocker (-63.2 ± 10.3 pA, *n* = 9). The amplitude of the NA-dependent inward current in the presence of the selective β_2_-adrenergic blocker ICI 118,551 (-58.5 ± 6.9 mV, *n* = 5, *p* = 0.1927, unpaired *t*-test, **Figures 5Da,b**) was not different from the amplitude of the current evoked by NA alone (-44.3 ± 7.3, *n* = 5).

**FIGURE 6:**
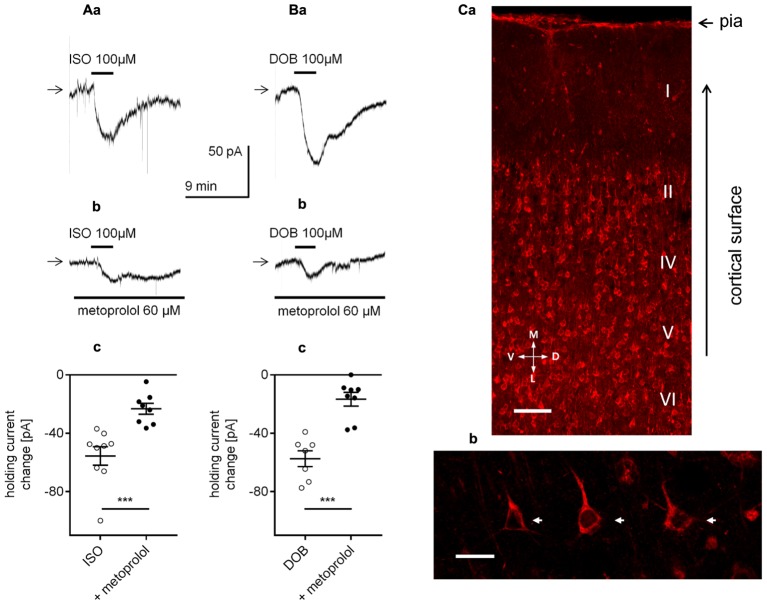
Effect of β_1_-adrenergic receptor stimulation on the holding current in layer V mPFC pyramidal neurons. **(A)** Inward currents evoked by bath application of the non-selective β-adrenergic receptor agonist isoproterenol alone (ISO, 100 μM, **a**) and isoproterenol (ISO, 100 μM) in the presence of the selective β_1_-adrenergic receptor blocker metoprolol (60 μM in the bath, **b**). Amplitude of the holding current change evoked by 100 μM isoproterenol alone (ISO) and 100 μM isoproterenol in the presence of 60 μM metoprolol (+ metoprolol, **c**). **(B)** Inward currents evoked by bath application of the selective β_1_-adrenergic receptor agonist dobutamine alone (DOB, 100 μM, **a**) and dobutamine (DOB, 100 μM) in the presence of the selective β_1_-adrenergic receptor blocker metoprolol (60 μM in the bath, **b**). Amplitude of the holding current change evoked by 100 μM dobutamine alone (DOB) and 100 μM dobutamine in the presence of 60 μM metoprolol (+ metoprolol, **c**); ^∗∗∗^*p* < 0.001. **(C)** Immunofluorescent staining of β_1_-adrenergic receptor protein in the rat mPFC. The signal is localized to neurons in different cortical layers **(a)**. Layer V at higher magnification with a pyramidal neuron showing immunofluorescent signal within its soma (arrows) and apical dendrites **(b)**. Scale bars **(a)** 100 μm, **(b)** 25 μm. M, medial; L, lateral; D, dorsal; V, ventral.

Next, we determined whether the stimulation of β-adrenergic receptors mimicked the effect of NA on the holding current. The currents were recorded in the classical whole-cell configuration. Application of a non-selective β-receptor agonist isoproterenol (100 μM, Kobayashi, 2007; Meitzen et al., 2011; Bateman et al., 2012) or a selective β_1_-receptor agonist dobutamine (100 μM, Bateman et al., 2012; Oshima et al., 2014) evoked significant changes in the holding current (-55.6 ± 6.4, *n* = 9, *p* < 0.0001, one-sample *t*-test, **Figure 6Aa** for isoproterenol and -57.4 ± 5.4 pA, *n* = 7, *p* < 0.0001, one-sample *t*-test, **Figure 6Ba** for dobutamine), which were markedly diminished in the presence of the β_1_-adrenergic receptor blocker metoprolol (60 μM, **Figures 6Ab,Bb**). The isoproterenol-dependent inward current in the presence of metoprolol decreased to -23.2 ± 3.8 pA (*n* = 8) and was significantly smaller than the control current (isoproterenol in the absence of metoprolol, -55.6 ± 6.4, *n* = 9, *p* = 0.0007, unpaired *t*-test, **Figure 6Ac**). Similarly, the dobutamine-dependent inward current in the presence of metoprolol was lower (-16.7 ± 4.7 pA, *n* = 8) than the inward current amplitude evoked by dobutamine alone (-57.4 ± 5.4 pA, *n* = 7, *p* < 0.0001, unpaired *t*-test, **Figure 6Bc**). Thus, the NA-dependent depolarization and inward current mainly depend on the activation of the β_1_-adrenergic receptor.

β_1_-adrenergic receptors have previously been shown to be expressed in mPFC neurons (Montezinho et al., 2006). Here, β_1_-adrenergic receptors were demonstrated to be located in layer V mPFC pyramidal neurons (**Figures 6Ca,b**).

Membrane potential recordings performed in the perforated-patch configuration strongly suggested that the membrane potential of mPFC pyramidal neurons is controlled by β_1_- but not by α_1_- or α_2_-adrenergic receptors. The β_1_-adrenergic receptor-dependent depolarization was mirrored by an inward current. Since the signal-to-noise ratio in the current recordings was superior to that in membrane potential recordings, the effects of the biologically active compounds were tested on the holding current in the remaining experiments. Currents were recorded in the classical whole-cell configuration, as the effects of adrenergic receptor stimulation obtained in the perforated-patch and classical whole-cell configurations were demonstrated to be similar (**Figures 2B,C**). Moreover, the classical whole-cell configuration enabled intracellular application of transduction system inhibitors within the pipette solution.

### Identification of the Cellular Effector Responsible for the Inward Current Evoked by β_1_-Adrenergic Receptor Stimulation

**FIGURE 7:**
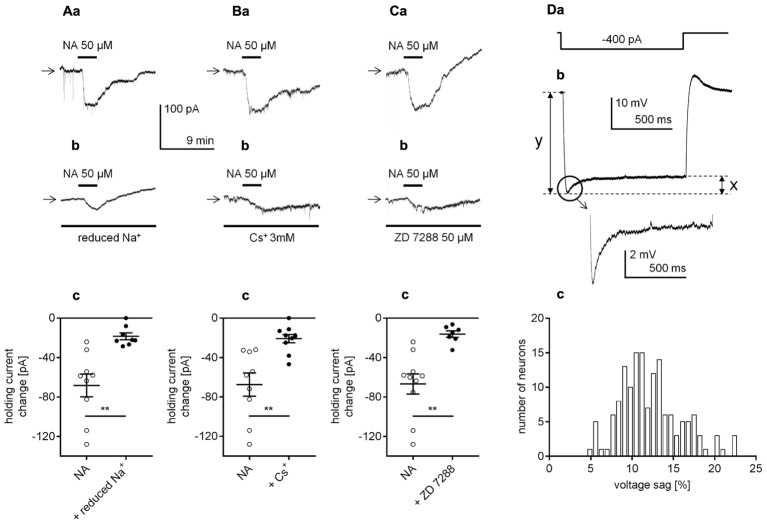
Identification of the cellular effector responsible for the β_1_-adrenergic receptor-dependent inward current in layer V mPFC pyramidal neurons. **(A)** Inward currents evoked by bath application of NA alone (50 μM, **a**) and by NA (50 μM) in the presence of a reduced Na^+^ concentration in the extracellular solution **(b)**. Amplitude of the holding current change evoked by 50 μM NA alone (NA) and 50 μM NA in the presence of a reduced extracellular Na^+^ concentration (+ reduced Na^+^, **c**). **(B)** Inward currents evoked by bath application of NA alone (50 μM, **a**) and by NA (50 μM) in the presence of Cs^+^ (3 mM in the bath, **b**). Amplitude of the holding current change evoked by 50 μM NA alone (NA) and 50 μM NA in the presence of 3 mM Cs^+^ (+ Cs^+^, **c**). **(C)** Inward currents evoked by bath application of NA alone (50 μM, **a**) and by NA (50 μM) in the presence of ZD 7288 (50 μM in the bath, **b**). Amplitude of the holding current change evoked by 50 μM NA alone (NA) and 50 μM NA in the presence of 50 μM ZD 7288 (+ ZD 7288, **c**); ^∗∗^*p* < 0.01. **(D)** The membrane potential change **(b)** evoked by rectangular negative current steps (-400 pA, lasting 1000 ms, **a**). Amplified voltage sag shown in the inset. Histogram of the voltage sag size [%] in layer V mPFC pyramidal neurons **(c)**. The size of the voltage sag was calculated by the difference between the maximum amplitude and sustained current response (*x* in **Db**) as a percentage of the maximum current response (*y* in **Db**).

The β_1_-related inward current may depend on the opening of Na^+^ channels, resulting in the influx of Na^+^ ions into the cytoplasm. To verify this possibility, we tested whether the amplitude of the β_1_-adrenergic receptor-dependent inward current decreased after a reduction in the extracellular Na^+^ concentration. Replacing Na^+^ (125 mM from 151.25 mM) with choline (125 mM) in the extracellular solution hyperpolarized the membrane potential (-72.6 ± 1.0, *n* = 22) when compared to the mean resting membrane potential in the control conditions (-66.0 ± 0.8 mV, *n* = 15, *p* < 0.0001, unpaired *t*-test, data not shown). The amplitude of the NA-related inward current was significantly smaller (-18.5 ± 3.5 pA, *n* = 8, *p* = 0.0013, unpaired *t*-test, **Figures 7Ab,c**) in the reduced extracellular Na^+^ concentration compared with the amplitude in the control conditions (-68.3 ± 11.5 pA, *n* = 9, **Figures 7Aa,c**). Thus, activation of the β_1_-adrenergic receptor evoked a Na^+^-dependent inward current.

Hyperpolarization-activated cyclic nucleotide-gated channels, which are permeable to both Na^+^ and K^+^ ions, are abundant in mPFC pyramidal neurons (for example Wang et al., 2007; Paspalas et al., 2013). Moreover, the membrane potential and holding current of these neurons are controlled by constitutively active HCN channels (Pisani et al., 2003; Rosenkranz and Johnston, 2006; Li et al., 2010; Nakashima et al., 2013; Cordeiro Matos et al., 2015). To test the involvement of HCN channels in the β_1_-dependent inward current, we applied NA in the presence of HCN channel blockers (Cs^+^ and ZD 7288). When Cs^+^ (3 mM), a non-selective HCN channel blocker (DiFrancesco, 1982; Carr et al., 2007), was added to the extracellular solution, the membrane potential hyperpolarized (-69.8 ± 0.8, *n* = 16) comparing to the mean resting membrane potential without Cs^+^ in the bath (-65.5 ± 0.5 mV, *n* = 16, *p* < 0.0001, unpaired *t*-test, data not shown). Once a stable membrane potential level was established, NA was applied to the bath. In the presence of Cs^+^ the NA-dependent inward current was decreased to -20.8 ± 4.2 pA (*n* = 10, *p* = 0.0012, unpaired *t*-test, **Figures 7Bb,c**), compared to the current in control conditions (-67.4 ± 11.7, *n* = 9, **Figures 7Ba,c**). When ZD 7288 (50 μM), a selective HCN channel blocker was added to the bath, the membrane potential was hyperpolarized (**Figure 4Da**). In the presence of ZD 7288 the amplitude of the inward current evoked by NA was also greatly reduced (-16.2 ± 3.3 pA, *n* = 7, *p* = 0.0012, unpaired *t*-test, **Figures 7Cb,c**) compared with that of the control (-66.8 ± 10.2, *n* = 10, **Figures 7Ca,c**).

Similar results were obtained when the inward current was evoked by bath application of the selective β_1_-adrenergic receptor agonist dobutamine (data not shown). The amplitude of the inward current evoked by dobutamine (100 μM) was significantly decreased from -45.2 ± 4.7 pA (*n* = 5) in the control condition to -20.8 ± 2.4 pA (*n* = 8, *p* = 0.0004, unpaired *t*-test) in the reduced Na^+^ condition. In the presence of Cs^+^ ions (3 mM) or ZD 7288 (50 μM) in the bath, the amplitude of the dobutamine-dependent inward current decreased from -50.8 ± 6.8 (*n* = 6) to -7.8 ± 2.5 pA (*n* = 6, *p* = 0.0001, unpaired *t*-test) in the presence of Cs^+^ ions and from -41.3 ± 4.1 (*n* = 7) to -16.3 ± 3.4 pA (*n* = 7, *p* = 0.0005, unpaired *t*-test) in the presence of ZD 7288.

Next, we wanted to test whether HCN channels were present in layer V mPFC pyramidal neurons in our experimental conditions. The expression of the HCN channel current in neurons is documented by the presence of a voltage sag evoked by a negative current step (Li et al., 2010; Cordeiro Matos et al., 2015; Gamo et al., 2015; Van Aerde and Feldmeyer, 2015). To evoke the voltage sag, a hyperpolarizing rectangular current step was applied (-400 pA, 1000 ms applied every 7 s, **Figure 7Da**). Importantly, the voltage sag was present in all tested layer V mPFC pyramidal neurons (**Figure 7Db** and inset). The size of the voltage sag was expressed by the difference between the maximum current amplitude and the sustained current response (line “*x*” in **Figure 7Db**) as a percentage of the maximum current response (line “*y*” in **Figure 7Db**) (compare Van Aerde and Feldmeyer, 2015; Van Aerde et al., 2015). The mean voltage sag size [%] was 12.2 ± 0.3% (M ± SE, *n* = 150). In **Figure 7Dc**, a histogram of the voltage sag size calculated for layer V mPFC pyramidal neurons is shown.

In conclusion, stimulation of the β_1_-adrenergic receptor evoked a Na^+^-dependent inward current flowing through HCN channels in mPFC pyramidal neurons.

### Identification of the Cellular Transduction System Responsible for the Inward Current Evoked by β_1_-Adrenergic Receptor Stimulation

**FIGURE 8:**
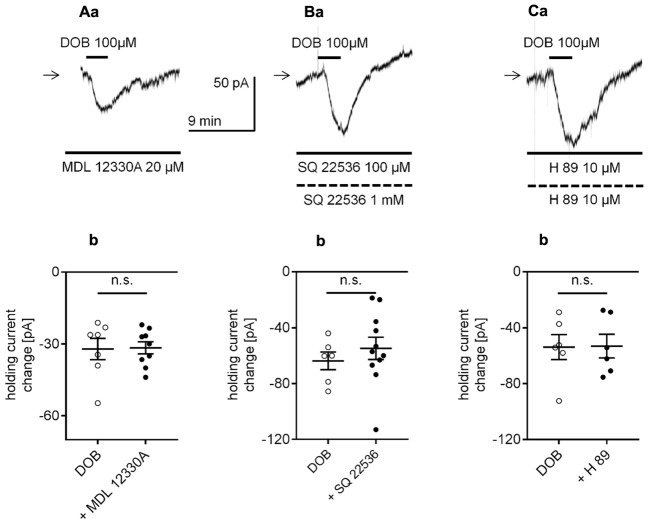
Effect of the adenylyl cyclase (AC) and protein kinase A (PKA) inhibitors on the β_1_-adrenergic receptor-dependent inward current in layer V mPFC pyramidal neurons. **(A)** Inward current evoked by bath application of dobutamine (DOB, 100 μM) in the presence of the AC inhibitor (MDL 12330A, 20 μM in the bath **a**). Amplitude of the holding current change evoked by 100 μM dobutamine alone (DOB) and 100 μM dobutamine in the presence of 20 μM MDL 12330A (+ MDL 12330A, **b**). **(B)** Inward current evoked by bath application of dobutamine (DOB, 100 μM) in the presence of the AC inhibitor (SQ 22536, 100 μM in the bath, 1 mM in the pipette, **a**). Amplitude of the holding current change evoked by 100 μM dobutamine alone (DOB) and 100 μM dobutamine in the presence of 100 μM SQ 22536 in the bath and 1 mM in the pipette (+ SQ 22536, **b**). **(C)** Inward current evoked by bath application of dobutamine (DOB, 100 μM) in the presence of the PKA inhibitor (H 89, 10 μM in the bath and 10 μM in the pipette, **a**). Amplitude of the holding current change evoked by 100 μM dobutamine alone (DOB) and 100 μM dobutamine in the presence of 10 μM H 89 in the bath and 10 μM in the pipette (+ H 89, **b**); n.s., non-significant. Broken horizontal bars below the recording traces indicate the pipette/intracellular solution presence of the compounds in this and other figures.

β_1_-adrenergic receptors are typically linked to the adenylyl cyclase/cAMP/protein kinase A (PKA) transduction pathway (Kobayashi, 2007; Meitzen et al., 2011). Therefore, we first examined whether adenylyl cyclase (AC) and PKA were involved in the observed β_1_-adrenergic-dependent inward current. Extracellular application of the AC inhibitor MDL 12330A (20 μM, Wang et al., 2008; Zhang et al., 2009; Simons et al., 2012; Socodato et al., 2017) did not affect the amplitude of the dobutamine-dependent inward current. The dobutamine-dependent inward current amplitudes without and in the presence of the inhibitor in the bath were -32.11 ± 4.5 pA (n = 7) and -31.6 ± 2.5 pA (n = 9), respectively (*p* = 0.9172, unpaired t-test, **Figures 8Aa,b**). The effect of dobutamine on the holding current was also tested in the presence of another membrane-permeable AC inhibitor (SQ 22536, 100 μM in the bath, 1 mM in the pipette solution, Rosenkranz and Johnston, 2006; Carr et al., 2007; Gu et al., 2007). SQ 22536 did not change the amplitude of the inward current (-54.8 ± 8 pA, *n* = 11, *p* = 0.4646, unpaired t-test, **Figures 8Ba,b**) compared to control (-63.8 ± 6.4 pA, *n* = 6). Next, in the presence of a membrane-permeable PKA inhibitor (H 89, 10 μM in the bath and pipette solution, Yi et al., 2013; Cordeiro Matos et al., 2015), the amplitude of the dobutamine-induced inward current was also unaltered (-53.2 ± 8.5 pA, *n* = 6, *p* = 0.9558, unpaired t-test compared to control -53.9 ± 8.9 pA, *n* = 6, **Figures 8Ca,b**). Neither MDL 12330A (*n* = 9, *p* = 0.2752), SQ 22536 (*n* = 11, *p* = 0.1363) nor H 89 (*n* = 12, *p* = 0.9627) significantly changed the membrane potential level when applied alone (data not shown).

**FIGURE 9:**
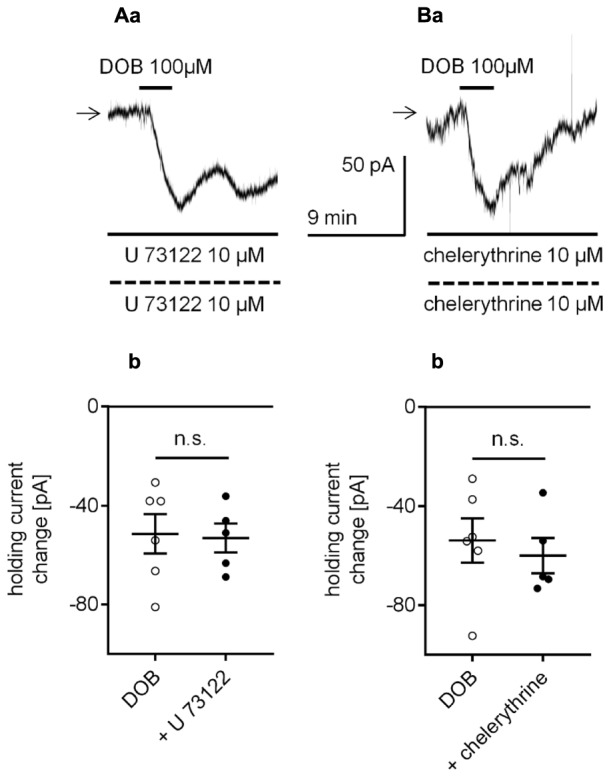
Effect of the phospholipase C (PLC) and protein kinase C (PKC) inhibitors on the β_1_-adrenergic receptor-dependent inward current in layer V mPFC pyramidal neurons. **(A)** Inward current evoked by bath application of dobutamine (DOB, 100 μM) in the presence of the PLC inhibitor (U 73122, 10 μM in the bath and 10 μM in the pipette, **a**). Amplitude of the holding current change evoked by 100 μM dobutamine alone (DOB) and 100 μM dobutamine in the presence of 10 μM U 73122 in the bath and 10 μM in the pipette (+ U 73122, **b**). **(B)** Inward current evoked by bath application of dobutamine (DOB, 100 μM) in the presence of the PKC inhibitor (chelerythrine, 10 μM in the bath and 10 μM in the pipette, **a**). Amplitude of the holding current change evoked by 100 μM dobutamine alone (DOB) and 100 μM dobutamine in the presence of 10 μM chelerythrine in the bath and 10 μM in the pipette (+ chelerythrine, **b**); n.s., non-significant.

Adrenergic receptors can also control cellular effectors via activation of the transduction system linked to phospholipase C (PLC) and protein kinase C (PKC) (Kobayashi, 2007; Luo et al., 2014). To test this option, we first applied a membrane-permeable PLC inhibitor (U 7322) to the bath (10 μM) and pipette (10 μM) solution. During administration of U 7322, the dobutamine-dependent current amplitude was -53.1 ± 5.9 pA (*n* = 5, **Figures 9Aa,b**) and was not different from the control current amplitude (-51.4 ± 7.9, *n* = 6, *p* = 0.8737, unpaired t-test). Application of the PKC inhibitor (chelerythrine) to the bath (10 μM) and pipette (10 μM) solution similarly did not affect the dobutamine-induced inward current. The dobutamine-dependent current amplitude in the presence of chelerythrine was -60.0 ± 7.1 pA (*n* = 5, **Figures 9Ba,b**) and was not significantly different from the inward current measured in control conditions when dobutamine alone was applied to the bath (-53.9 ± 9.0, *n* = 6, *p* = 0.6171, unpaired t-test). Neither of the inhibitors (U 7322, *n* = 13, *p* = 0.8677; chelerythrine, *n* = 10, *p* = 0.9024) had a significant effect on the resting membrane potential when applied alone (data not shown).

**FIGURE 10:**
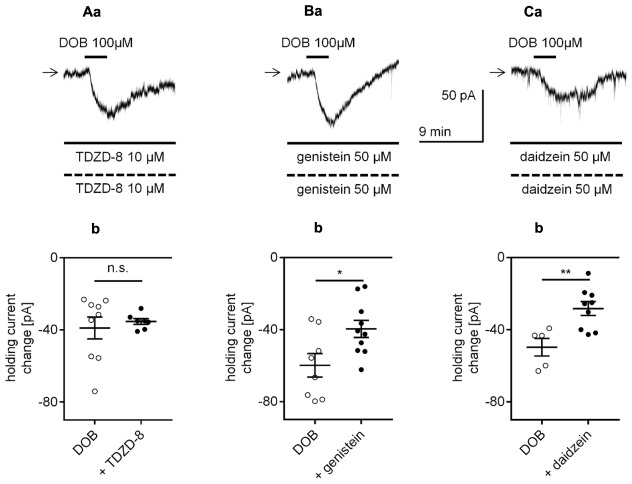
Effects of glycogen synthase kinase-3β (GSK-3β) and tyrosine kinase inhibitors on the β_1_-adrenergic receptor-dependent inward current in layer V mPFC pyramidal neurons. **(A)** Inward current evoked by bath application of dobutamine (DOB, 100 μM) in the presence of the GSK-3β inhibitor (TDZD-8, 10 μM in the bath and 10 μM in the pipette, **a**). Amplitude of the holding current change evoked by 100 μM dobutamine alone (DOB) and 100 μM dobutamine in the presence of 10 μM TDZD-8 in the bath and 10 μM in the pipette (+ TDZD-8, **b**). **(B)** Inward current evoked by bath application of dobutamine (DOB, 100 μM) in the presence of the tyrosine kinase inhibitor (genistein, 50 μM in the bath and 50 μM in the pipette, **a**). Amplitude of the holding current change evoked by 100 μM dobutamine alone (DOB) and 100 μM dobutamine in the presence of 50 μM genistein in the bath and 50 μM in the pipette (+ genistein, **b**). **(C)** Inward current evoked by bath application of dobutamine (DOB, 100 μM) in the presence of an inactive analog of genistein (daidzein, 50 μM in the bath and 50 μM in the pipette, **a**). Amplitude of the holding current change evoked by 100 μM dobutamine alone (DOB) and 100 μM dobutamine in the presence of 50 μM daidzein in the bath and 50 μM in the pipette (+ daidzein, **b**); ^∗^*p* < 0.05; ^∗∗^*p* < 0.01; n.s., non-significant.

Glycogen synthase kinase-3β (GSK-3β) may be involved in the signal transduction from catecholamine receptors to cellular effectors in mPFC pyramidal neurons (Li et al., 2009; Xing et al., 2016). Nevertheless, in our study, the continuous presence of the GSK-3β inhibitor TDZD-8 (10 μM in the bath and 10 μM in the pipette solution, Li et al., 2009, 2013) did not significantly change the amplitude of the dobutamine-dependent inward current compared with that of the control (evoked by application of dobutamine alone). When dobutamine was applied alone (100 μM) and together with TDZD-8 (**Figures 10Aa,b**), the amplitudes of the inward currents were not significantly different (-38.9 ± 6.0 pA, *n* = 9 and -35.3 ± 1.6 pA, *n* = 7, respectively, *p* = 0.6139, unpaired *t*-test). Application of TDZD-8 alone did not affect the membrane potential level (*n* = 7, *p* = 0.3647, data not shown).

Catecholamines can modulate cellular effectors via activation of tyrosine kinase (Gao and Wolf, 2008). In our study, in the presence of genistein, a tyrosine kinase inhibitor (50 μM in the bath and 50 μM in the pipette solution), the amplitude of the inward current evoked by bath application of dobutamine (100 μM, -39.6 ± 4.7 pA, *n* = 10, **Figures 10Ba,b**) was significantly lower than that of the inward current evoked by application of dobutamine alone (100 μM, -59.8 ± 6.5 pA, *n* = 8, *p* = 0.0210, unpaired *t*-test). However, application of daidzein (50 μM in the bath and 50 μM in the pipette solution, Wong et al., 2010), an inactive analog of genistein, also diminished the dobutamine-induced inward current (-28.3 ± 3.8, *n* = 9, *p* = 0.0053, unpaired *t*-test, **Figures 10Ca,b**) compared with the inward current evoked by application of dobutamine alone (-49.7 ± 4.9, *n* = 5). The membrane potential was not changed in the presence of any of the inhibitors alone (genistein, *n* = 10, *p* = 0.8612; daidzein, *n* = 9, *p* = 0.5404, data not shown). Therefore, genistein decreased the current amplitude via its non-specific action and not via inhibition of tyrosine kinase receptors (Wong et al., 2010).

The obtained results suggest that the AC/PKA, PLC/PKC, GSK-3β and tyrosine kinase signaling pathways are not involved in the signal transduction between β_1_-adrenergic receptors and HCN channels.

**FIGURE 11:**
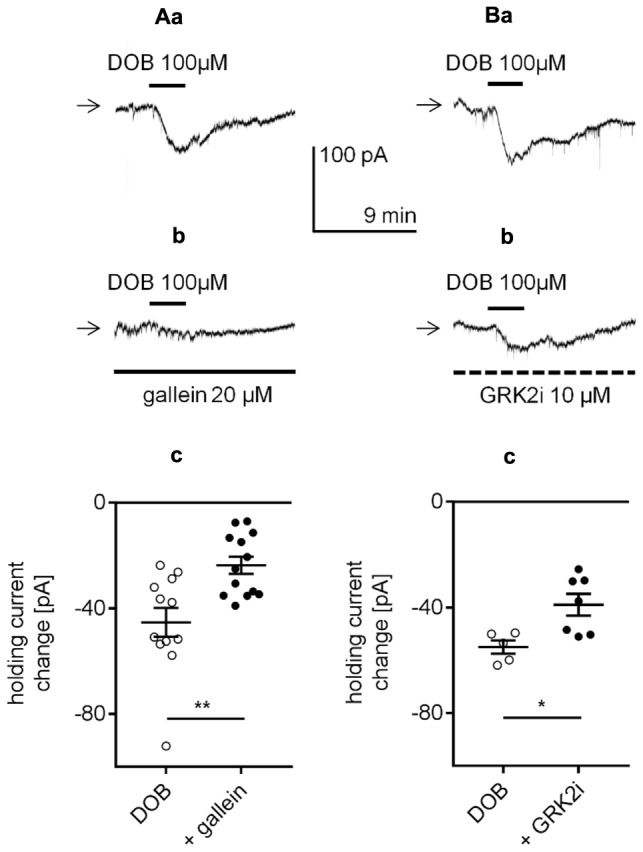
Effect of βγ subunit-dependent signaling inhibitors on the β_1_-adrenergic receptor-dependent inward current in layer V mPFC pyramidal neurons. **(A)** Inward currents evoked by bath application of dobutamine alone (DOB, 100 μM, **a**) and dobutamine (DOB, 100 μM) in the presence of gallein (20 μM in the bath, **b**). Amplitude of the holding current change evoked by 100 μM dobutamine alone (DOB) and 100 μM dobutamine in the presence of 20 μM gallein (+ gallein, **c**). The slices were also exposed to gallein in the extracellular solution for 2 h before current recordings. **(B)** Inward currents evoked by bath application of dobutamine alone (DOB, 100 μM, **a**) and dobutamine (DOB, 100 μM) in the presence of GRK2i (10 μM in the pipette solution, **b**). Amplitude of the holding current change evoked by 100 μM dobutamine in the absence (DOB) and presence of GRK2i 10 μM (+ GRK2i) **(c)**. The currents were recorded >50 min after obtaining access to the cell; ^∗^*p* < 0.05, ^∗∗^*p* < 0.01.

In this study, the amplitude of the β_1_-adrenergic-dependent inward current and membrane depolarization was not significantly different when recordings were performed in either the classical whole-cell configuration or perforated-patch configuration. If cytoplasmic second messengers were involved, then the amplitude of the β_1_-dependent changes in the membrane potential and holding current would be smaller when recorded in the classical whole-cell configuration due to “dialysis” of second messengers from the cell. However, the results presented in **Figure 2** demonstrate that the β_1_-dependent depolarization and inward current amplitudes did not differ between recordings in the perforated-patch and classical whole-cell configuration. For this reason and since the classical transduction systems were not involved (see above), we presumed that signal transduction might occur in a membrane-delimited fashion involving the G-protein βγ subunit as this transduction system is less sensitive to the absence of cytoplasmic second messengers (Dascal, 2001; Hatcher-Solis et al., 2014). We investigated this possibility using three tests that block βγ signaling in different ways. First, the brain slices were incubated with gallein (20 μM) for >2 h (Belkouch et al., 2011; Meitzen et al., 2011; Ukhanov et al., 2011; Kurowski et al., 2015). Gallein belongs to a class of small molecules that block the transduction system involving the Gβγ subunit (Lehmann et al., 2008; Smrcka, 2013). In the presence of gallein in the extracellular solution, the amplitude of the dobutamine-dependent inward current was -23.7 ± 3.3 pA (*n* = 13, **Figures 11Ab,c**), which was significantly smaller than the current amplitude evoked in the absence of gallein (-45.3 ± 5.5 pA, *n* = 12, *p* = 0.0022, unpaired *t*-test, **Figures 11Aa,c**).

Second, the effect of GRK2i, a Gβγ antagonist polypeptide that inhibits the activation of G-protein-coupled receptor kinase 2 (GRK2, Diverse-Pierluissi et al., 1996; Dang et al., 2009; Stott et al., 2015), was tested. The neurons were “dialyzed” with GRK2i (10 μM) via the recording pipette for >50 min. Dobutamine (100 μM) was added to the bath 50 min after the recording pipette with or without GRK2i obtained access to the cell. In the presence of GRK2i in the pipette solution, the dobutamine-dependent inward current was significantly smaller (-39.0 ± 4.1 pA, *n* = 7, *p* = 0.0136, unpaired *t*-test, **Figures 11Bb,c**) than the control current (-55.0 ± 2.5 pA, *n* = 5, **Figures 11Ba,c**) when GRK2i was absent from the pipette solution. Neither of the inhibitors (gallein, *n* = 13, *p* = 0.4479; GRK2i, *n* = 7, *p* = 0.6262) had a significant effect on the resting membrane potential when applied alone (data not shown).

**FIGURE 12:**
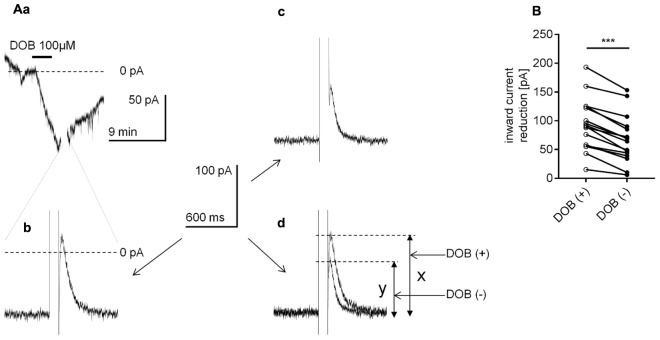
Effect of the voltage step on the β_1_-adrenergic receptor-dependent inward current in layer V mPFC pyramidal neurons. **(A)** Inward current evoked by bath application of dobutamine alone (DOB, 100 μM, **a**). At the peak of the inward current, the recording was interrupted, and a 100-ms depolarizing voltage step from the holding potential to +80 mV was applied. Following the voltage step, the inward current was reduced **(b)**. At 20 min after termination of dobutamine application, when the recorded holding current returned to its resting control level, a current was injected from the pipette to obtain the maximum inward current level similar to that during the application of dobutamine. At this current level, a 100-ms depolarizing voltage step from the holding potential to +80 mV was applied again. Following the voltage step, the inward current was reduced **(c)**. In **(d)**, the overlapped current recordings from **(b,c)** are shown. The amplitude of the current reduction in the presence (*x*) and absence (*y*) of dobutamine is indicated. **(B)** Amplitude of the inward current reduction after the voltage step during dobutamine application [DOB (+)] and in the absence of dobutamine [DOB (–)] in the extracellular solution; ^∗∗∗^*p* < 0.001.

A large voltage step disrupts the binding of the Gβγ subunit to its membrane effector, leading to an attenuation of the signal transduction from metabotropic receptors to membrane effectors if the transduction occurs in a membrane-delimited fashion (Zamponi and Snutch, 1998; Currie, 2010). To investigate this mechanism, at the peak of the dobutamine-dependent inward current (**Figure 12Aa**), we applied a 100-ms depolarizing voltage step of approximately 150 mV. Directly after the voltage step, the inward current was abolished and moved slightly above the 0 current level in 16 of the 18 tested neurons (**Figure 12Ab**, 0 current level indicated by the broken line). After 15–20 min, when the current returned to the level before dobutamine bath application, we injected an inward current to obtain the level attained at the peak inward current during β_1_-agonist application. The voltage step (150 mV, 100 ms) was applied again and diminished this artificially evoked inward current (**Figure 12Ac**). To compare the effect of the voltage steps on the reduction of the inward current in the presence and absence of dobutamine, we overlapped the traces shown in (b) and (c) (**Figure 12Ad**). The reduction in the current amplitude after the voltage step in the presence of dobutamine was 93.3 ± 11.2 pA (*n* = 16, “*x*” in **Figure 12Ad**), whereas the reduction in the current amplitude in the absence of dobutamine was significantly smaller (*p* < 0.0001, paired *t*-test, **Figure 12B**) at 67.6 ± 10.4 pA (*n* = 16, “*y*” in **Figure 12Ad**). Thus, the voltage step may disconnect the Gβγ subunit to a greater extent in the presence than in the absence of dobutamine in the bath.

## Discussion

In this study, we examined the effect of adrenergic receptor stimulation on the membrane potential and holding current in layer V mPFC pyramidal neurons in slices. Application of NA evoked depolarization or an inward current in the tested neurons irrespective of whether the recordings were performed in the classical whole-cell or perforated-patch configuration. NA increased the number of action potentials evoked by the same depolarizing current step. The effect of NA depended on β_1_- but not α_1_- or α_2_-adrenergic receptor stimulation. Activation of β_1_-adrenergic receptors increased the inward Na^+^ current flowing through HCN channels, which are permeable to Na^+^ and K^+^ ions. Here, we demonstrated that the transduction system most likely operated in a membrane-delimited fashion and involved the βγ subunit of G-protein.

Presumably, NA released from the adrenergic terminals diffuses throughout the interstitial fluid and stimulates adrenergic receptors located on multiple cells in the mPFC (Fuxe et al., 2015). Therefore, NA may directly alter layer V pyramidal neuron activity by acting on adrenergic receptors expressed in these cells or indirectly by modulating the activity of neurons with synaptic contacts with pyramidal neurons. Indirect effects have been demonstrated; for example, NA can affect the GABAergic and glutamatergic transmission that control mPFC pyramidal neuron activity (Kawaguchi and Shindou, 1998; Kobayashi et al., 2009; Wang et al., 2013; Zhang et al., 2013). To avoid indirect effects of NA on the tested pyramidal neurons in the slices, in a great majority of the recordings, we synaptically isolated the neurons by bath application of GABAergic and glutamatergic blockers and a voltage-gated Na^+^ channel blocker, TTX. In these experimental conditions, spontaneous and evoked excitatory and inhibitory postsynaptic potentials and action potentials were absent in the tested pyramidal neurons.

### The Adrenergic Receptor Responsible for the Noradrenaline-Dependent Depolarization and Inward Current in mPFC Pyramidal Neurons

The effects of NA on the membrane potential are cell type-specific (Kawaguchi and Shindou, 1998). For example, NA depolarizes cholinergic interneurons in the striatum (Pisani et al., 2003), depolarizes or hyperpolarizes spinal dorsal horn neurons (Gassner et al., 2009), and hyperpolarizes hypocretin neurons in the hypothalamus (Li and van den Pol, 2005). In this study, application of NA invariably evoked depolarization or an inward current in mPFC pyramidal neurons.

NA can potentially activate all classes of adrenergic receptors (α_1_, α_2_, and β) to control membrane potential levels in mPFC pyramidal neurons. Stimulation of postsynaptic α_1_-adrenergic receptors causes increased excitability (Wang and McCormick, 1993), elevated (Luo et al., 2014) or suppressed (Kobayashi, 2007; Kobayashi et al., 2009) glutamatergic excitatory postsynaptic potentials (EPSPs) amplitude and suppressed long-term depression in rat mPFC pyramidal neurons (Marzo et al., 2010). In turn, stimulation of α_2_-adrenergic receptors by NA evokes hyperpolarization and increased excitability (Carr et al., 2007), persistent firing (Zhang et al., 2013), inhibits glutamatergic EPSPs (Ji et al., 2008; Yi et al., 2013) or long-term depression (Marzo et al., 2010) in mPFC pyramidal neurons. The reported effects of β-adrenergic receptor stimulation have been more consistent. Stimulation of β-adrenergic receptors on mPFC pyramidal neurons evokes depolarization, increased excitability (Mueller et al., 2008), increased glutamatergic EPSP frequency and amplitude (Kobayashi, 2007; Ji et al., 2008) and enhanced long-term potentiation (Zhou et al., 2013).

In the present study, application of two different α_1_-adrenergic receptor agonists, phenylephrine and cirazoline, at doses typically used in other studies (Imbery et al., 2008; Luo et al., 2014) failed to mimic the NA-dependent effect on the membrane potential. Similarly, stimulation of the α_2_-adrenergic receptor with two different agonists, medetomidine and clonidine, did not evoke the depolarization observed during application of NA. Thus, NA may not modulate the resting membrane potential level through α-type adrenergic receptors.

However, the amplitudes of the NA-dependent depolarization and inward current were decreased in the presence of the selective β_1_-adrenergic receptor blocker metoprolol. The effects of NA were not impacted by the selective β_2_-adrenergic receptor blocker ICI 118,551. Moreover, application of isoproterenol, a non-selective β-adrenergic receptor agonist, and dobutamine, a selective β_1_-adrenergic receptor agonist (Bateman et al., 2012), mimicked the NA-dependent inward current. The inward current evoked by application of the two β-receptor agonists was also significantly diminished in the presence of the β_1_-receptor blocker metoprolol. These results indicate that NA preferentially modulates the membrane potential and holding current in mPFC pyramidal neurons through β_1_-adrenergic receptors. Furthermore, using immunofluorescence, we showed the presence of β_1_-adrenergic receptors in mPFC pyramidal neurons in rats, which supports the earlier results of Montezinho et al. (2006) who found these receptors in mPFC cells. The residual depolarization and inward current evoked by application of NA in the presence of the selective β_1_-adrenergic receptor blocker might be attributed to an incomplete blockade of β_1_-adrenergic receptors by metoprolol or stimulation of other β-adrenergic receptor subtypes, such as β_3_-adrenergic receptors (Claustre et al., 2008; Ursino et al., 2009), potentially present in the PFC.

Sierra-Mercado et al. (2011) indicated that layer V mPFC pyramidal neurons in prelimbic and infralimbic areas have different functions. In our study, application of adrenergic receptor agonists (NA, dobutamine or isoproterenol) evoked depolarization or an inward current in 283 pyramidal neurons randomly chosen from both areas (this study and Grzelka and Szulczyk unpublished results). Therefore, we presume that pyramidal neurons from both the prelimbic and infralimbic area are likewise controlled by adrenergic receptors.

Our findings agree with the findings reported by Wang and McCormick (1993) and Mueller et al. (2008) in which NA evoked depolarization and an increase in excitability of mPFC pyramidal neurons. Nevertheless, Wang and McCormick (1993) reported that NA modulated excitability and the membrane potential through stimulation of α_1_- and not β-adrenergic receptors. Our results are inconsistent with the studies conducted by Carr et al. (2007), which reported that stimulation of α_2_-adrenergic receptors evoked hyperpolarization and an increase in pyramidal neuron excitability in mPFC pyramidal neurons.

### Ionic Mechanism Responsible for the β_1_-Dependent Inward Current in mPFC Pyramidal Neurons

β_1_-adrenergic receptor-dependent changes in the resting membrane potential and holding current reflect the same physiological process, i.e., increased inward current in mPFC pyramidal neurons. Compared with recordings of the membrane potential, current recordings achieved a superior signal-to-noise ratio. For this reason, the current recordings were used to discern the detailed mechanism responsible for the β_1_-adrenergic receptor-related control of inward current.

The properties of the β_1_-dependent inward current described in this study suggest that it depends on channels that are constitutively active, have a threshold close to the membrane potential and do not become inactivated or only slightly inactivated over time.

The NA-related inward current was markedly diminished when the Na^+^ ion concentration in the extracellular solution was reduced, suggesting involvement of Na^+^ ions in the β_1_-dependent inward current and depolarization. HCN channels are controlled by β_1_-adrenergic receptors in the heart (Brown et al., 1979; DiFrancesco, 1995). In other studies, activation of β-adrenergic receptors evoked depolarization by shifting the voltage dependence of HCN activation to more depolarized potentials, for example, in striatal cholinergic interneurons (Pisani et al., 2003), hippocampal stratum oriens-alveus interneurons (Maccaferri and McBain, 1996), cerebellar basket cells (Saitow and Konishi, 2000) and olfactory receptor neurons (Nakashima et al., 2013). Moreover, HCN channels are present in mPFC pyramidal neurons (Wang et al., 2007; Paspalas et al., 2013). Evidence from electrophysiological (Magee, 1998; Williams and Stuart, 2000; Atkinson and Williams, 2009) and immunostaining (Lörincz et al., 2002; Atkinson and Williams, 2009) methods has shown that HCN channels increase in density from the soma to the apical dendrites in pyramidal neurons (for review: Ramaswamy and Markram, 2015). To investigate the involvement of HCN channels, we tested the effects of bath application of non-specific (Cs^+^, DiFrancesco, 1982) and specific (ZD 7288, Gasparini and DiFrancesco, 1997; Day et al., 2005; Li et al., 2010; for review: Biel et al., 2009) blockers of HCN channels on the β_1_-adrenergic-dependent inward current. Both blockers markedly inhibited the inward current evoked by NA as well as by dobutamine application in mPFC pyramidal neurons.

The expression of the HCN channel current in neurons is documented by the presence of a voltage sag evoked by a rectangular negative current step (Li et al., 2010; Cordeiro Matos et al., 2015; Gamo et al., 2015; Van Aerde and Feldmeyer, 2015). In some layer V mPFC pyramidal neurons, the voltage sag is absent, suggesting that these neurons do not express HCN channels (Dembrow et al., 2010). However, other studies have indicated that the voltage sag can be evoked in all layer V mPFC pyramidal neurons and that the voltage sag was absent in some pyramidal neurons located just outside of layer V (Van Aerde and Feldmeyer, 2015; Van Aerde et al., 2015). Furthermore, others did not report the absence of HCN channels in layer V mPFC pyramidal neurons (Magee, 1998; Williams and Stuart, 2000; Berger et al., 2001; Lörincz et al., 2002; Day et al., 2005; Wang et al., 2007; Atkinson and Williams, 2009; Li et al., 2010; Ramaswamy and Markram, 2015). In agreement with the findings of Van Aerde and Feldmeyer (2015), Van Aerde et al. (2015) the voltage sag could be evoked in all tested pyramidal neurons in this study, suggesting that all layer V mPFC pyramidal neurons express HCN channels.

A residual β_1_-dependent inward current remained in the presence of HCN channel blockers. This residual current could have been due to an incomplete blockade of the HCN channels or another ionic conductance involved in the membrane potential control by β_1_-adrenergic receptors. Furthermore, this current may depend on activation of a TTX-resistant Na^+^ current (Kurowski et al., 2015; Gawlak et al., 2017; Radzicki et al., 2017) or inhibition of constitutively active K^+^ currents (Ładno et al., 2017).

Altogether, the obtained results indicate that HCN channel activation greatly contributes to the observed NA-dependent inward current similarly to other cell types.

### The Signal Transduction System Responsible for the β_1_-Dependent Inward Current in mPFC Pyramidal Neurons

Metabotropic adrenergic receptors are coupled to G-protein and transduce their effects via different cellular signaling pathways. β-adrenergic receptors are linked to the AC/cAMP/PKA signal transduction system (Benovic, 2002; Ursino et al., 2009). In agreement with this finding, stimulation of β_1_-adrenergic receptors in mPFC pyramidal neurons evokes cellular effects mediated by the cAMP/PKA cascade (Kobayashi, 2007; Ji et al., 2008; Ładno et al., 2017). However, in the present study, selective blockers of AC (MDL 12330A or SQ 22536) and PKA (H 89) applied at concentrations used in other studies (Carr et al., 2007; Gu et al., 2007; Zhang et al., 2009; Yi et al., 2013; Socodato et al., 2017) failed to affect the β_1_-related inward current, indicating that β_1_-adrenergic receptors control the holding current in pyramidal neurons through the cAMP/PKA independent pathway.

The PLC/PKC signaling system is linked to adrenergic receptors in mPFC neurons (Kobayashi, 2007; Luo et al., 2014). However, the amplitude of the β_1_-related inward current was not altered in the presence of selective blockers of PLC (U 7322) or PKC (chelerythrine).

In other studies, catecholamines, including NA, modulate prefrontal cortex functions through pathways involving GSK-3β (Li et al., 2009; Xing et al., 2016). Nevertheless, application of the selective inhibitor of GSK-3β, TDZD-8, at a concentration used in other studies (Li et al., 2009) did not decrease the β_1_-related inward current in our study.

Catecholamines can elicit their action in mPFC neurons via tyrosine kinase (Gao and Wolf, 2008). Although genistein, a tyrosine kinase blocker, decreased the β_1_-related inward current, the effect was also diminished in the presence of its inactive analog daidzein (Zong et al., 2005; Wong et al., 2010). This finding indicates that the effect of genistein was probably due to its non-specific action and not due to selective inhibition of tyrosine kinase.

The perforated-patch method avoids the washout of cytoplasmic second messengers potentially involved in signal transduction from adrenergic receptors to the cellular effector. Interestingly, applying the classical whole-cell method, which occurs with cell membrane rupture and may cause “dialysis” of cytoplasmic messengers from neurons, did not diminish the effects of NA on the tested neurons. However, the amplitudes of the adrenergic-dependent depolarization and inward current were not significantly different between recordings performed in the classical whole-cell configuration and those performed using the perforated-patch method. Thus, cytoplasmic second messengers might not be involved in the transduction system. Considering this finding and that all tested inhibitors of typical cytoplasmic second messenger systems failed to inhibit the β_1_-induced inward current, we considered the involvement of a cytoplasmic-independent transduction system. Adrenergic receptors can control cellular effectors, e.g., N-type Ca^++^ channels (Delmas et al., 1999), MAP kinase (Daaka et al., 1997), angiotensin AT1-receptors (Talaia et al., 2006), AMPA receptors (Yuen et al., 2014), K^+^ channels (Kodama and Togari, 2013), and G-protein-coupled receptor kinase 2 (Cannavo et al., 2013), in a membrane-delimited fashion via the βγ subunit in both neuronal and non-neuronal cells. To a great extent, this signal transduction is independent of cytoplasmic second messengers (Dascal, 2001; Hatcher-Solis et al., 2014).

In the present study, extracellular application of gallein, a small molecule inhibitor that prevents interaction of the βγ subunit with the effector (Lehmann et al., 2008; Smrcka, 2013; Kurowski et al., 2015), reduced the amplitude of the β_1_-related inward current. The β_1_-adrenergic receptor stimulation was also attenuated by perfusion of the neuron with the GRK2i polypeptide. GRK2i inhibits activation of G-protein-coupled receptor kinase 2 by the βγ subunit (Diverse-Pierluissi et al., 1996; Dang et al., 2009; Stott et al., 2015). Moreover, a large amplitude, depolarizing voltage step abolished the inward current evoked by application of dobutamine, indicating a disconnection between the βγ subunit and controlled ion channel.

Hyperpolarization-activated cyclic nucleotide-gated channels can be directly controlled by cAMP (DiFrancesco and Tortora, 1991; Ludwig et al., 1998; Santoro et al., 1998; Wainger et al., 2001) and by transduction systems linked to phospholipase C (for review: Suh and Hille, 2005; Pian et al., 2007) and protein kinase A pathways (Vargas and Lucero, 2002; Cheng and Zhou, 2013). Our results suggest that β_1_-adrenergic receptors modulate HCN channels in mPFC pyramidal neurons via the βγ subunit of G-protein.

### Functional Significance of mPFC Pyramidal Neurons Control by NA

NA released from adrenergic terminals can stimulate adrenergic receptors conceivably located on multiple cells present in the prefrontal cortex and can postsynaptically and presynaptically control pyramidal neurons through volume transmission (Fuxe et al., 2015).

Activation of presynaptic β-adrenergic receptors located on glutamatergic terminals increases EPSP and excitatory postsynaptic current (EPSC) amplitude in mPFC pyramidal neurons by facilitating glutamate release (Ji et al., 2008; Kobayashi et al., 2009). Leak-type TREK K^+^ currents can be inhibited by the stimulation of postsynaptic β-adrenergic receptors in mPFC pyramidal neurons (Ładno et al., 2017). This inhibition may promote depolarization of pyramidal neurons. Moreover, activation of postsynaptic β-adrenergic receptors can increase the availability of voltage-dependent Na^+^ currents (Szulczyk, 2015), which lowers the threshold for action potential generation. The present study shows that NA acting through β_1_-adrenergic receptors in mPFC pyramidal neurons results in depolarization due to activation of the inward Na^+^ current through HCN channels. Therefore, pre- and postsynaptic activation of β_1_-adrenergic receptors may support “up-states,” which appear as a prolonged depolarization and persistent activity at the depolarization peak in mPFC pyramidal neurons and reflect the working memory process (O’Donnell, 2008).

## Conclusion

Our study provides a mechanism for the direct excitatory effects of extracellular application of NA on mPFC pyramidal neurons. The involvement of β_1_-adrenergic receptors, HCN channels and the βγ subunit in the NA-induced depolarization and inward current leads to a better understanding of NA-mediated mPFC activity.

## Author Contributions

KG performed the experiments and analyzed data. PK and MG performed some experiments and discussed the research. KG and PS designed the experiments, prepared figures and wrote the article.

## Conflict of Interest Statement

The authors declare that the research was conducted in the absence of any commercial or financial relationships that could be construed as a potential conflict of interest.
